# (μ-4,4′-Bipyridine-κ^2^ *N*:*N*′)bis­[triaqua­(4,4′-bipyridine-κ*N*)(3-nitro­phthalato-κ*O* ^2^)cobalt(II)]

**DOI:** 10.1107/S160053680902875X

**Published:** 2009-07-25

**Authors:** Hong-Xu Guo, Zhong-Liang Yao, Wen Weng, Xi-Zhong Li

**Affiliations:** a Department of Chemistry and Environmental Science, Zhangzhou Normal University, Zhangzhou, Fujian 363000, People’s Republic of China; b Department of Biology and Chemical Engineering, Fuqing Branch of Fujian Normal University, Fuqing, Fujian 350300, People’s Republic of China

## Abstract

The title binuclear complex, [Co_2_(C_8_H_3_NO_6_)_2_(C_10_H_8_N_2_)_3_(H_2_O)_6_], has been synthesized hydro­thermally from 3-nitro­phthalic acid (H_2_NPA), Co(NO_3_)_2_·6H_2_O and 4,4′-bipyridine (4,4′-bipy). The mol­ecule of the complex occupies a special position on an inversion centre. The Co^II^ atom has a slightly distorted octa­hedral environment formed by two N atoms from two 4,4′-bipy ligands, one carboxyl­ate O atom from NPA, and three O atoms of water mol­ecules. An extensive O—H⋯O and N—H⋯O hydrogen-bonding system links mol­ecules of the complex into a three-dimensional network.

## Related literature

For background to metal-involved supra­molecular compounds, see: Noro (2004 ▶); Yaghi *et al.* (2003 ▶); Rao *et al.* (2004 ▶); Huang *et al.* (2004 ▶); Zhang *et al.* (2004 ▶). For other 3-nitro­phthalic derivatives, see: Deng *et al.* (2007 ▶); Guo (2004 ▶); Song *et al.* (2007 ▶); Xiong & Qi (2007 ▶).
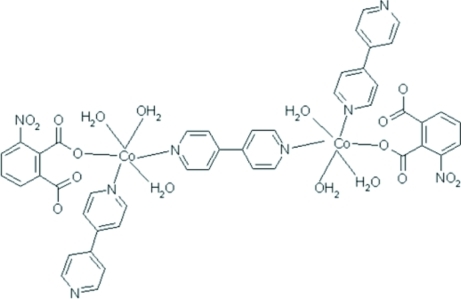


## Experimental

### 

#### Crystal data

[Co_2_(C_8_H_3_NO_6_)_2_(C_10_H_8_N_2_)_3_(H_2_O)_6_]
*M* *_r_* = 1112.74
Monoclinic, 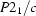

*a* = 15.672 (3) Å
*b* = 9.4283 (19) Å
*c* = 16.063 (3) Å
β = 103.92 (3)°
*V* = 2303.8 (8) Å^3^
*Z* = 2
Mo *K*α radiation
μ = 0.81 mm^−1^
*T* = 293 K
0.21 × 0.15 × 0.12 mm

#### Data collection

Siemens SMART CCD area-detector diffractometer
Absorption correction: multi-scan (*SADABS*; Sheldrick, 1996 ▶) *T*_min_ = 0.765, *T*_max_ = 0.872
21789 measured reflections
5252 independent reflections
3669 reflections with *I* > 2σ(*I*)
*R*_int_ = 0.079

#### Refinement

*R*[*F* ^2^ > 2σ(*F* ^2^)] = 0.051
*wR*(*F* ^2^) = 0.134
*S* = 1.01
5252 reflections
352 parameters
9 restraints
H atoms treated by a mixture of independent and constrained refinement
Δρ_max_ = 0.38 e Å^−3^
Δρ_min_ = −0.45 e Å^−3^

### 

Data collection: *SMART* (Siemens, 1994 ▶); cell refinement: *SAINT* (Siemens, 1994 ▶); data reduction: *SAINT*; program(s) used to solve structure: *SHELXTL* (Sheldrick, 2008 ▶); program(s) used to refine structure: *SHELXTL*; molecular graphics: *SHELXTL*; software used to prepare material for publication: *SHELXTL*.

## Supplementary Material

Crystal structure: contains datablocks I, global. DOI: 10.1107/S160053680902875X/ya2101sup1.cif

Structure factors: contains datablocks I. DOI: 10.1107/S160053680902875X/ya2101Isup2.hkl

Additional supplementary materials:  crystallographic information; 3D view; checkCIF report

## Figures and Tables

**Table 1:** Hydrogen-bond geometry (Å, °)

*D*—H⋯*A*	*D*—H	H⋯*A*	*D*⋯*A*	*D*—H⋯*A*
O7—H7*A*⋯O3^i^	0.842 (10)	1.960 (11)	2.798 (3)	173 (3)
O7—H7*B*⋯O4^ii^	0.845 (10)	1.942 (12)	2.772 (3)	167 (3)
O8—H8*A*⋯O2	0.854 (10)	1.855 (13)	2.677 (3)	161 (3)
O8—H8*B*⋯N3^iii^	0.847 (10)	2.021 (15)	2.830 (4)	159 (3)
O9—H9*B*⋯O4^i^	0.849 (10)	1.810 (13)	2.645 (3)	167 (3)
O9—H9*C*⋯O3	0.849 (10)	1.968 (13)	2.801 (3)	167 (3)

Symmetry codes: (i) 
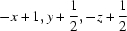
; (ii) 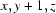; (iii) 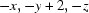.

**Fig. 1.**
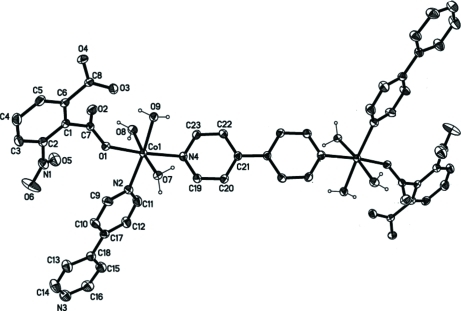
Molecular structure of the title compound, showing the atom-labelling scheme. Displacement ellipsoids are drawn at the 35% probability level; H-atoms bound to phenyl C atoms are omitted for clarity. H-atoms bound to O are shown as small circles of arbitrary radius. The unlabeled atoms are derived from their labeled counterparts via symmetry transformation (-x + 1,-y + 2,-z + 1).

**Fig. 2.**
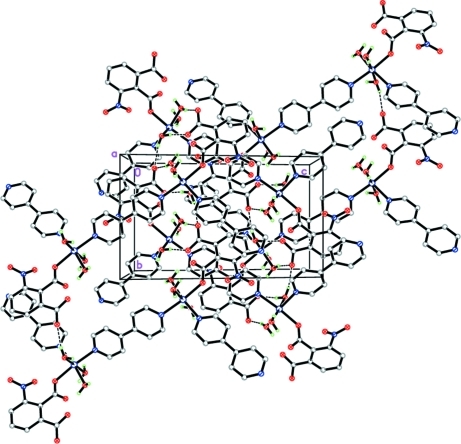
Crystal packing of the title compound viewed down the a axis; H-bonds are shown as dashed lines.

## supplementary crystallographic information

### Comment

Design and assembly of metal-involving supramolecular architectures are currently of great interest in the field of supramolecular chemistry and crystal engineering, because they can provide novel topology and functional materials (Noro, 2004; Yaghi *et al.*, 2003; Rao *et al.*, 2004). During the past decades, extensive efforts have been focused on the design and assembly of supramolecular architectures of this kind (Huang *et al.*, 2004; Zhang *et al.*, 2004). Although the multifunctional ligand, 3-nitrophthalic acid (H_2_NPA), has been utilized to build many coordination complexes, such as dinuclear centrosymmetric complexes [LaL(HL)(H_2_O)_3_]_2_.2H_2_O (*L* = NPA) (Deng *et al.*, 2007), [La_2_(C_8_H_3_NO_6_)_2_(C_8_H_4_NO_6_)_2_(H_2_O)_6_].2H_2_O (Xiong & Qi, 2007), and [Na(C_8_H_4_NO_6_)(H_2_O)_3_].H_2_O (Guo, 2004), only a few mixed ligand complexes involving NPA have been reported so far (Song *et al.*, 2007). In this work, we employed NPA and 4,4'-bipy ligands to produce a novel binuclear complex, [Co_2_(NPA)_2_(bipy)_3_(H_2_O)_6_](I).

Complex (I) occupies a special position in the inversion centre; the asymmetric unit consists of one cobalt(II) atom, one NPA ligand, one terminal and one-half of a bridging 4,4'-bipy groups, as well as three metal-coordinated water molecules.(Fig. 1 and Table 1). The Co1 atom has a a slightly distorted octahedral environment formed by two N atoms from two different bipy ligands, one carboyxlate O atom of the NPA ligand, and three water molecules. The µ_2_-4,4'-bipyridine ligand bridges two [Co(NPA)(bipy)(H_2_O)_3_] units of the binuclear complex.

The extensive system of O—H···O hydrogen bonds links molecules of the complex into a three-dimensional network (Fig. 2; Table 1).

### Experimental

A solution of Co(NO_3_)_2_.6H_2_O (0.0291 g, 0.1 mmol) in 5 ml of water was added dropwise under continuous stirring to an aqueous solution (5 ml) of 3-nitrophthalic acid (0.0211 g, 0.1 mmol) and 4,4'-bipyridine (0.0156 g, 0.1 mmol). The resulting mixture was then transferred into a Teflon-lined stainless steel vessel, which was sealed and kept at 393 K for 72 h. The vessel was then cooled to room temperature, the reaction mixture was filtered, and single crystals were obtained from the filtrate after a few days of slow evaporation at room temperature.

### Refinement

The aromatic H atoms were positioned geometrically and allowed to ride during subsequent refinement, with C—H = 0.93 Å and *U*_iso_(H) = 1.2*U*_eq_(C). Water H atoms were located in a difference map and refined with O—H and H···H distance restraints of 0.85 (1) and 1.39 (1) Å, respectively and *U*_iso_(H)= 1.2*U*_eq_(O).

### Crystal data

**Table:**

[Co_2_(C_8_H_3_NO_6_)_2_(C_10_H_8_N_2_)_3_(H_2_O)_6_]	*F*(000) = 1144
*M**_r_* = 1112.74	*D*_x_ = 1.604 Mg m^−^^3^
Monoclinic, *P*2_1_/*c*	Mo *K*α radiation, λ = 0.71073 Å
Hall symbol: -P 2ybc	Cell parameters from 21789 reflections
*a* = 15.672 (3) Å	θ = 3.0–27.5°
*b* = 9.4283 (19) Å	µ = 0.81 mm^−^^1^
*c* = 16.063 (3) Å	*T* = 293 K
β = 103.92 (3)°	Prism, pink
*V* = 2303.8 (8) Å^3^	0.21 × 0.15 × 0.12 mm
*Z* = 2	

### Data collection

**Table:**

Siemens SMART CCD area-detector diffractometer	5252 independent reflections
Radiation source: fine-focus sealed tube	3669 reflections with *I* > 2σ(*I*)
graphite	*R*_int_ = 0.079
Detector resolution: no pixels mm^-1^	θ_max_ = 27.5°, θ_min_ = 3.0°
ω scans	*h* = −20→20
Absorption correction: multi-scan (*SADABS*; Sheldrick, 1996)	*k* = −12→12
*T*_min_ = 0.765, *T*_max_ = 0.872	*l* = −20→19
21789 measured reflections	

### Refinement

**Table:**

Refinement on *F*^2^	Primary atom site location: structure-invariant direct methods
Least-squares matrix: full	Secondary atom site location: difference Fourier map
*R*[*F*^2^ > 2σ(*F*^2^)] = 0.051	Hydrogen site location: inferred from neighbouring sites
*wR*(*F*^2^) = 0.134	H atoms treated by a mixture of independent and constrained refinement
*S* = 1.01	*w* = 1/[σ^2^(*F*_o_^2^) + (0.075*P*)^2^] where *P* = (*F*_o_^2^ + 2*F*_c_^2^)/3
5252 reflections	(Δ/σ)_max_ = 0.001
352 parameters	Δρ_max_ = 0.38 e Å^−^^3^
9 restraints	Δρ_min_ = −0.45 e Å^−^^3^

### Special details

**Table:**

Geometry. All e.s.d.'s (except the e.s.d. in the dihedral angle between two l.s. planes) are estimated using the full covariance matrix. The cell e.s.d.'s are taken into account individually in the estimation of e.s.d.'s in distances, angles and torsion angles; correlations between e.s.d.'s in cell parameters are only used when they are defined by crystal symmetry. An approximate (isotropic) treatment of cell e.s.d.'s is used for estimating e.s.d.'s involving l.s. planes.
Refinement. Refinement of *F*^2^ against ALL reflections. The weighted *R*-factor *wR* and goodness of fit *S* are based on *F*^2^, conventional *R*-factors *R* are based on *F*, with *F* set to zero for negative *F*^2^. The threshold expression of *F*^2^ > σ(*F*^2^) is used only for calculating *R*-factors(gt) *etc*. and is not relevant to the choice of reflections for refinement. *R*-factors based on *F*^2^ are statistically about twice as large as those based on *F*, and *R*- factors based on ALL data will be even larger.

### Fractional atomic coordinates and isotropic or equivalent isotropic displacement parameters (Å^2^)

**Table:**

	*x*	*y*	*z*	*U*_iso_*/*U*_eq_	
Co1	0.32172 (2)	0.70261 (4)	0.20771 (2)	0.02477 (14)	
N1	0.17512 (19)	0.4939 (3)	−0.07320 (18)	0.0434 (7)	
N2	0.24471 (16)	0.8547 (3)	0.12309 (15)	0.0299 (6)	
N3	−0.0484 (2)	1.2759 (4)	−0.1856 (2)	0.0507 (8)	
N4	0.35953 (16)	0.8342 (3)	0.31977 (14)	0.0288 (6)	
O1	0.30328 (14)	0.5593 (2)	0.10689 (12)	0.0303 (5)	
O2	0.22712 (15)	0.3866 (2)	0.15197 (13)	0.0398 (6)	
O3	0.41953 (15)	0.2894 (2)	0.18540 (13)	0.0378 (6)	
O4	0.43538 (15)	0.0753 (2)	0.13428 (14)	0.0395 (6)	
O5	0.13514 (18)	0.5266 (3)	−0.02137 (16)	0.0565 (7)	
O6	0.1662 (2)	0.5543 (4)	−0.14226 (19)	0.0909 (12)	
O7	0.43623 (14)	0.7886 (2)	0.17266 (13)	0.0308 (5)	
H7A	0.4781 (16)	0.782 (3)	0.2166 (14)	0.037*	
H7B	0.4312 (19)	0.8724 (16)	0.1535 (17)	0.037*	
O8	0.21785 (14)	0.6093 (3)	0.25287 (13)	0.0337 (5)	
H8A	0.2092 (19)	0.534 (2)	0.2224 (18)	0.040*	
H8B	0.1712 (13)	0.658 (3)	0.243 (2)	0.040*	
O9	0.40189 (14)	0.5401 (2)	0.27330 (13)	0.0331 (5)	
H9B	0.4550 (9)	0.560 (3)	0.2970 (18)	0.040*	
H9C	0.4003 (18)	0.469 (2)	0.2406 (17)	0.040*	
C1	0.28309 (19)	0.3469 (3)	0.02915 (18)	0.0272 (6)	
C2	0.23784 (19)	0.3751 (3)	−0.05515 (18)	0.0312 (7)	
C3	0.2485 (2)	0.2972 (4)	−0.1248 (2)	0.0386 (8)	
H3A	0.2170	0.3202	−0.1800	0.046*	
C4	0.3062 (2)	0.1854 (4)	−0.1112 (2)	0.0420 (8)	
H4A	0.3132	0.1298	−0.1570	0.050*	
C5	0.3537 (2)	0.1565 (3)	−0.02903 (19)	0.0359 (7)	
H5A	0.3936	0.0817	−0.0203	0.043*	
C6	0.34391 (19)	0.2355 (3)	0.04132 (18)	0.0283 (7)	
C7	0.26975 (19)	0.4381 (3)	0.10318 (17)	0.0273 (6)	
C8	0.4034 (2)	0.1983 (3)	0.12786 (19)	0.0290 (6)	
C9	0.2360 (2)	0.8381 (4)	0.03926 (19)	0.0380 (8)	
H9A	0.2676	0.7659	0.0210	0.046*	
C10	0.1829 (2)	0.9217 (4)	−0.02198 (19)	0.0378 (8)	
H10A	0.1795	0.9052	−0.0798	0.045*	
C11	0.1994 (2)	0.9611 (4)	0.1467 (2)	0.0401 (8)	
H11A	0.2049	0.9761	0.2050	0.048*	
C12	0.1452 (2)	1.0492 (4)	0.0896 (2)	0.0400 (8)	
H12A	0.1155	1.1221	0.1096	0.048*	
C13	0.0546 (2)	1.0864 (4)	−0.1498 (2)	0.0481 (9)	
H13A	0.0827	1.0105	−0.1690	0.058*	
C14	−0.0048 (3)	1.1679 (5)	−0.2077 (2)	0.0558 (11)	
H14A	−0.0148	1.1454	−0.2656	0.067*	
C15	0.0285 (2)	1.2325 (4)	−0.0402 (2)	0.0442 (9)	
H15A	0.0388	1.2599	0.0170	0.053*	
C16	−0.0308 (3)	1.3064 (4)	−0.1027 (3)	0.0531 (10)	
H16A	−0.0603	1.3824	−0.0855	0.064*	
C17	0.13452 (19)	1.0300 (3)	0.00195 (19)	0.0300 (7)	
C18	0.07229 (19)	1.1180 (4)	−0.06323 (19)	0.0320 (7)	
C19	0.3917 (2)	0.9648 (3)	0.31517 (18)	0.0331 (7)	
H19A	0.3766	1.0121	0.2629	0.040*	
C20	0.4460 (2)	1.0327 (3)	0.38372 (18)	0.0310 (7)	
H20A	0.4674	1.1229	0.3769	0.037*	
C21	0.46875 (18)	0.9661 (3)	0.46269 (17)	0.0261 (6)	
C22	0.4311 (2)	0.8345 (3)	0.46904 (18)	0.0336 (7)	
H22A	0.4417	0.7880	0.5216	0.040*	
C23	0.3779 (2)	0.7729 (4)	0.39713 (18)	0.0340 (7)	
H23A	0.3536	0.6845	0.4027	0.041*	

### Atomic displacement parameters (Å^2^)

**Table:**

	*U*^11^	*U*^22^	*U*^33^	*U*^12^	*U*^13^	*U*^23^
Co1	0.0295 (2)	0.0217 (2)	0.0215 (2)	−0.00056 (17)	0.00289 (16)	−0.00077 (15)
N1	0.0390 (16)	0.0472 (19)	0.0376 (15)	0.0047 (14)	−0.0032 (14)	0.0032 (14)
N2	0.0317 (13)	0.0290 (15)	0.0277 (12)	0.0015 (11)	0.0045 (11)	0.0015 (11)
N3	0.0410 (17)	0.055 (2)	0.0513 (18)	0.0092 (15)	0.0009 (15)	0.0066 (15)
N4	0.0353 (14)	0.0283 (15)	0.0223 (11)	−0.0019 (11)	0.0062 (11)	−0.0032 (10)
O1	0.0412 (12)	0.0224 (12)	0.0254 (10)	−0.0036 (9)	0.0044 (9)	−0.0011 (8)
O2	0.0486 (14)	0.0332 (14)	0.0409 (12)	−0.0088 (11)	0.0170 (11)	−0.0025 (10)
O3	0.0447 (13)	0.0299 (13)	0.0323 (11)	0.0056 (10)	−0.0036 (10)	−0.0036 (9)
O4	0.0456 (13)	0.0205 (12)	0.0458 (13)	0.0039 (10)	−0.0020 (10)	0.0026 (9)
O5	0.0578 (16)	0.0590 (19)	0.0494 (15)	0.0215 (14)	0.0065 (13)	−0.0057 (13)
O6	0.103 (3)	0.112 (3)	0.0584 (18)	0.053 (2)	0.0208 (17)	0.0473 (19)
O7	0.0360 (12)	0.0228 (12)	0.0312 (11)	−0.0017 (9)	0.0032 (9)	0.0009 (9)
O8	0.0325 (11)	0.0340 (13)	0.0335 (11)	0.0033 (10)	0.0060 (10)	0.0027 (9)
O9	0.0364 (12)	0.0256 (12)	0.0321 (11)	0.0023 (9)	−0.0017 (10)	−0.0028 (9)
C1	0.0291 (15)	0.0228 (16)	0.0275 (14)	−0.0066 (12)	0.0026 (12)	−0.0011 (12)
C2	0.0311 (16)	0.0282 (18)	0.0309 (15)	−0.0014 (13)	0.0008 (13)	0.0008 (13)
C3	0.0413 (18)	0.045 (2)	0.0258 (15)	−0.0057 (16)	0.0006 (14)	−0.0006 (14)
C4	0.051 (2)	0.045 (2)	0.0306 (16)	−0.0083 (17)	0.0111 (15)	−0.0097 (15)
C5	0.0416 (18)	0.0237 (17)	0.0419 (18)	−0.0010 (14)	0.0091 (15)	−0.0062 (14)
C6	0.0312 (16)	0.0218 (16)	0.0310 (15)	−0.0052 (12)	0.0054 (13)	0.0008 (12)
C7	0.0299 (15)	0.0229 (16)	0.0248 (14)	0.0017 (12)	−0.0020 (12)	−0.0007 (11)
C8	0.0307 (15)	0.0221 (16)	0.0320 (15)	−0.0039 (13)	0.0034 (13)	0.0023 (13)
C9	0.052 (2)	0.0327 (19)	0.0304 (16)	0.0115 (15)	0.0128 (15)	0.0041 (14)
C10	0.0468 (19)	0.041 (2)	0.0246 (15)	0.0107 (16)	0.0062 (14)	0.0040 (14)
C11	0.0445 (19)	0.042 (2)	0.0309 (16)	0.0059 (16)	0.0044 (15)	−0.0068 (15)
C12	0.0460 (19)	0.037 (2)	0.0340 (16)	0.0140 (16)	0.0048 (15)	−0.0081 (14)
C13	0.044 (2)	0.054 (2)	0.0421 (19)	0.0178 (17)	0.0011 (16)	−0.0041 (17)
C14	0.049 (2)	0.073 (3)	0.0392 (19)	0.020 (2)	−0.0007 (18)	−0.0034 (19)
C15	0.048 (2)	0.042 (2)	0.0410 (19)	0.0125 (17)	0.0076 (16)	0.0008 (16)
C16	0.056 (2)	0.042 (2)	0.060 (2)	0.0207 (18)	0.012 (2)	0.0046 (18)
C17	0.0271 (15)	0.0279 (17)	0.0345 (16)	0.0007 (13)	0.0062 (13)	0.0043 (13)
C18	0.0262 (15)	0.0337 (18)	0.0348 (16)	0.0024 (13)	0.0050 (13)	0.0026 (13)
C19	0.0444 (18)	0.0284 (18)	0.0246 (14)	−0.0008 (14)	0.0046 (13)	−0.0019 (12)
C20	0.0388 (17)	0.0259 (17)	0.0275 (14)	−0.0041 (13)	0.0061 (13)	−0.0022 (12)
C21	0.0292 (14)	0.0260 (16)	0.0232 (13)	−0.0017 (12)	0.0064 (12)	−0.0039 (12)
C22	0.0441 (18)	0.0344 (19)	0.0216 (14)	−0.0050 (14)	0.0065 (13)	0.0015 (13)
C23	0.0416 (18)	0.0325 (19)	0.0275 (15)	−0.0122 (14)	0.0077 (14)	−0.0028 (13)

### Geometric parameters (Å, °)

**Table:**

Co1—O1	2.075 (2)	C4—C5	1.378 (4)
Co1—O9	2.097 (2)	C4—H4A	0.9300
Co1—O8	2.126 (2)	C5—C6	1.393 (4)
Co1—N2	2.138 (2)	C5—H5A	0.9300
Co1—N4	2.149 (2)	C6—C8	1.517 (4)
Co1—O7	2.164 (2)	C9—C10	1.374 (4)
N1—O5	1.197 (4)	C9—H9A	0.9300
N1—O6	1.225 (4)	C10—C17	1.380 (4)
N1—C2	1.472 (4)	C10—H10A	0.9300
N2—C9	1.330 (4)	C11—C12	1.370 (4)
N2—C11	1.336 (4)	C11—H11A	0.9300
N3—C14	1.322 (5)	C12—C17	1.388 (4)
N3—C16	1.325 (5)	C12—H12A	0.9300
N4—C23	1.337 (4)	C13—C14	1.380 (5)
N4—C19	1.339 (4)	C13—C18	1.384 (4)
O1—C7	1.253 (4)	C13—H13A	0.9300
O2—C7	1.244 (4)	C14—H14A	0.9300
O3—C8	1.242 (4)	C15—C18	1.376 (5)
O4—C8	1.257 (4)	C15—C16	1.382 (5)
O7—H7A	0.842 (10)	C15—H15A	0.9300
O7—H7B	0.845 (10)	C16—H16A	0.9300
O8—H8A	0.854 (10)	C17—C18	1.498 (4)
O8—H8B	0.847 (10)	C19—C20	1.377 (4)
O9—H9B	0.849 (10)	C19—H19A	0.9300
O9—H9C	0.849 (10)	C20—C21	1.383 (4)
C1—C2	1.395 (4)	C20—H20A	0.9300
C1—C6	1.401 (4)	C21—C22	1.388 (4)
C1—C7	1.522 (4)	C21—C21^i^	1.497 (5)
C2—C3	1.382 (4)	C22—C23	1.380 (4)
C3—C4	1.372 (5)	C22—H22A	0.9300
C3—H3A	0.9300	C23—H23A	0.9300
			
O1—Co1—O9	82.56 (8)	C1—C6—C8	123.3 (3)
O1—Co1—O8	91.30 (9)	O2—C7—O1	127.5 (3)
O9—Co1—O8	86.66 (9)	O2—C7—C1	117.9 (3)
O1—Co1—N2	89.37 (9)	O1—C7—C1	114.7 (3)
O9—Co1—N2	170.96 (9)	O3—C8—O4	124.8 (3)
O8—Co1—N2	97.64 (9)	O3—C8—C6	119.5 (3)
O1—Co1—N4	171.19 (9)	O4—C8—C6	115.7 (3)
O9—Co1—N4	89.42 (9)	N2—C9—C10	123.7 (3)
O8—Co1—N4	91.87 (9)	N2—C9—H9A	118.2
N2—Co1—N4	98.34 (10)	C10—C9—H9A	118.2
O1—Co1—O7	90.54 (8)	C9—C10—C17	120.3 (3)
O9—Co1—O7	88.29 (9)	C9—C10—H10A	119.9
O8—Co1—O7	174.37 (8)	C17—C10—H10A	119.9
N2—Co1—O7	87.70 (9)	N2—C11—C12	123.4 (3)
N4—Co1—O7	85.57 (9)	N2—C11—H11A	118.3
O5—N1—O6	123.1 (3)	C12—C11—H11A	118.3
O5—N1—C2	119.7 (3)	C11—C12—C17	120.3 (3)
O6—N1—C2	117.2 (3)	C11—C12—H12A	119.9
C9—N2—C11	116.3 (3)	C17—C12—H12A	119.9
C9—N2—Co1	118.0 (2)	C14—C13—C18	119.8 (3)
C11—N2—Co1	125.5 (2)	C14—C13—H13A	120.1
C14—N3—C16	116.1 (3)	C18—C13—H13A	120.1
C23—N4—C19	116.7 (3)	N3—C14—C13	123.8 (3)
C23—N4—Co1	118.9 (2)	N3—C14—H14A	118.1
C19—N4—Co1	121.1 (2)	C13—C14—H14A	118.1
C7—O1—Co1	127.50 (19)	C18—C15—C16	119.5 (3)
Co1—O7—H7A	106 (2)	C18—C15—H15A	120.3
Co1—O7—H7B	116 (2)	C16—C15—H15A	120.3
H7A—O7—H7B	110.9 (16)	N3—C16—C15	124.2 (4)
Co1—O8—H8A	100 (2)	N3—C16—H16A	117.9
Co1—O8—H8B	114 (2)	C15—C16—H16A	117.9
H8A—O8—H8B	109.6 (16)	C10—C17—C12	116.0 (3)
Co1—O9—H9B	118 (2)	C10—C17—C18	121.6 (3)
Co1—O9—H9C	110 (2)	C12—C17—C18	122.4 (3)
H9B—O9—H9C	109.4 (16)	C15—C18—C13	116.5 (3)
C2—C1—C6	116.7 (3)	C15—C18—C17	121.9 (3)
C2—C1—C7	121.2 (3)	C13—C18—C17	121.5 (3)
C6—C1—C7	122.1 (2)	N4—C19—C20	123.5 (3)
C3—C2—C1	123.5 (3)	N4—C19—H19A	118.2
C3—C2—N1	116.7 (3)	C20—C19—H19A	118.2
C1—C2—N1	119.7 (3)	C19—C20—C21	119.7 (3)
C4—C3—C2	118.9 (3)	C19—C20—H20A	120.1
C4—C3—H3A	120.6	C21—C20—H20A	120.1
C2—C3—H3A	120.6	C20—C21—C22	116.9 (3)
C3—C4—C5	119.2 (3)	C20—C21—C21^i^	121.0 (3)
C3—C4—H4A	120.4	C22—C21—C21^i^	122.0 (3)
C5—C4—H4A	120.4	C23—C22—C21	119.8 (3)
C4—C5—C6	122.1 (3)	C23—C22—H22A	120.1
C4—C5—H5A	118.9	C21—C22—H22A	120.1
C6—C5—H5A	118.9	N4—C23—C22	123.2 (3)
C5—C6—C1	119.5 (3)	N4—C23—H23A	118.4
C5—C6—C8	117.2 (3)	C22—C23—H23A	118.4
			
O1—Co1—N2—C9	−21.2 (2)	C2—C1—C7—O2	106.9 (3)
O8—Co1—N2—C9	−112.4 (2)	C6—C1—C7—O2	−76.2 (4)
N4—Co1—N2—C9	154.5 (2)	C2—C1—C7—O1	−72.5 (4)
O7—Co1—N2—C9	69.3 (2)	C6—C1—C7—O1	104.5 (3)
O1—Co1—N2—C11	154.2 (3)	C5—C6—C8—O3	155.8 (3)
O8—Co1—N2—C11	63.0 (3)	C1—C6—C8—O3	−21.8 (5)
N4—Co1—N2—C11	−30.1 (3)	C5—C6—C8—O4	−21.9 (4)
O7—Co1—N2—C11	−115.2 (3)	C1—C6—C8—O4	160.4 (3)
O9—Co1—N4—C23	−31.5 (2)	C11—N2—C9—C10	−1.1 (5)
O8—Co1—N4—C23	55.1 (2)	Co1—N2—C9—C10	174.7 (3)
N2—Co1—N4—C23	153.1 (2)	N2—C9—C10—C17	−0.1 (5)
O7—Co1—N4—C23	−119.9 (2)	C9—N2—C11—C12	0.9 (5)
O9—Co1—N4—C19	127.1 (2)	Co1—N2—C11—C12	−174.5 (3)
O8—Co1—N4—C19	−146.3 (2)	N2—C11—C12—C17	0.5 (6)
N2—Co1—N4—C19	−48.3 (3)	C16—N3—C14—C13	1.5 (7)
O7—Co1—N4—C19	38.7 (2)	C18—C13—C14—N3	−1.1 (7)
O9—Co1—O1—C7	62.0 (2)	C14—N3—C16—C15	−0.5 (6)
O8—Co1—O1—C7	−24.5 (2)	C18—C15—C16—N3	−1.0 (6)
N2—Co1—O1—C7	−122.1 (2)	C9—C10—C17—C12	1.5 (5)
O7—Co1—O1—C7	150.2 (2)	C9—C10—C17—C18	−176.6 (3)
C6—C1—C2—C3	1.9 (5)	C11—C12—C17—C10	−1.7 (5)
C7—C1—C2—C3	179.0 (3)	C11—C12—C17—C18	176.5 (3)
C6—C1—C2—N1	−177.9 (3)	C16—C15—C18—C13	1.4 (5)
C7—C1—C2—N1	−0.8 (4)	C16—C15—C18—C17	−177.4 (3)
O5—N1—C2—C3	148.7 (3)	C14—C13—C18—C15	−0.5 (6)
O6—N1—C2—C3	−30.8 (5)	C14—C13—C18—C17	178.4 (4)
O5—N1—C2—C1	−31.5 (5)	C10—C17—C18—C15	−175.3 (3)
O6—N1—C2—C1	149.0 (3)	C12—C17—C18—C15	6.7 (5)
C1—C2—C3—C4	0.2 (5)	C10—C17—C18—C13	5.9 (5)
N1—C2—C3—C4	−180.0 (3)	C12—C17—C18—C13	−172.1 (3)
C2—C3—C4—C5	−1.8 (5)	C23—N4—C19—C20	4.3 (5)
C3—C4—C5—C6	1.3 (5)	Co1—N4—C19—C20	−154.8 (3)
C4—C5—C6—C1	0.9 (5)	N4—C19—C20—C21	−1.0 (5)
C4—C5—C6—C8	−176.8 (3)	C19—C20—C21—C22	−3.0 (5)
C2—C1—C6—C5	−2.4 (4)	C19—C20—C21—C21^i^	176.9 (3)
C7—C1—C6—C5	−179.5 (3)	C20—C21—C22—C23	3.6 (5)
C2—C1—C6—C8	175.2 (3)	C21^i^—C21—C22—C23	−176.3 (3)
C7—C1—C6—C8	−1.9 (5)	C19—N4—C23—C22	−3.6 (5)
Co1—O1—C7—O2	13.2 (4)	Co1—N4—C23—C22	156.0 (3)
Co1—O1—C7—C1	−167.53 (18)	C21—C22—C23—N4	−0.3 (5)

Symmetry codes: (i) −*x*+1, −*y*+2, −*z*+1.

### Hydrogen-bond geometry (Å, °)

**Table:**

*D*—H···*A*	*D*—H	H···*A*	*D*···*A*	*D*—H···*A*
O7—H7A···O3^ii^	0.84 (1)	1.96 (1)	2.798 (3)	173 (3)
O7—H7B···O4^iii^	0.85 (1)	1.94 (1)	2.772 (3)	167 (3)
O8—H8A···O2	0.85 (1)	1.86 (1)	2.677 (3)	161 (3)
O8—H8B···N3^iv^	0.85 (1)	2.02 (2)	2.830 (4)	159 (3)
O9—H9B···O4^ii^	0.85 (1)	1.81 (1)	2.645 (3)	167 (3)
O9—H9C···O3	0.85 (1)	1.97 (1)	2.801 (3)	167 (3)

Symmetry codes: (ii) −*x*+1, *y*+1/2, −*z*+1/2; (iii) *x*, *y*+1, *z*; (iv) −*x*, −*y*+2, −*z*.
